# Underutilization of Guideline-Directed Medical Therapy in Heart Failure With Reduced Ejection Fraction: A Retrospective Study in Rural Appalachia

**DOI:** 10.7759/cureus.91467

**Published:** 2025-09-02

**Authors:** Koushik Sanku, Sanskrita Nemalikanti, Oluwatobi Adegbile, Sudeepthi Mekala, Priyanka Vatsavayi, Adam Willey, Ghaith Husari, Hadii M Mamudu, Venkata Vedantam

**Affiliations:** 1 Columbia University Division of Cardiology, Mount Sinai Medical Center, Miami Beach, USA; 2 Department of Internal Medicine, East Tennessee State University, Johnson City, USA; 3 College of Public Health, East Tennessee State University, Johnson City, USA; 4 Center for Cardiovascular Risk Research, East Tennessee State University, Johnson City, USA; 5 Department of Computing, East Tennessee State University, Johnson City, USA

**Keywords:** angiotensin-converting enzyme inhibitors (acei), angiotensin-ii receptor blocker (arb), angiotensin receptor-neprilysin inhibitor (arni), beta-blockers, chronic heart failure, guideline-directed medical therapy (gdmt), hfref, mineralocorticoid receptor antagonist, renin-angiotensin-aldosterone system (raas), sglt2-inhibitors

## Abstract

Introduction: Guideline-directed medical therapy (GDMT) for heart failure with reduced ejection fraction (HFrEF) includes a combination of angiotensin-converting enzyme inhibitors (ACEI)/angiotensin receptor blockers (ARB)/angiotensin receptor neprilysin inhibitors (ARNI), beta-blockers, mineralocorticoid receptor agonists (MRA), and sodium-glucose co-transporter 2 inhibitors (SGLT2i). Despite mortality benefits, implementation remains suboptimal.

Methods: This was a retrospective observational study conducted in three outpatient Internal Medicine and Cardiology clinics within the East Tennessee State University Health system. Adults aged ≥18 years with ejection fraction (EF) ≤40% and at least one outpatient follow-up between December 2022 and November 2023 were included. Patients with contraindications to GDMT, end-stage renal disease, or limited life expectancy were excluded. GDMT use and dosing (none, <50%, ≥50 to <100%, 100% target dose) for beta-blockers, ACEI/ARB/ARNI, MRA, and SGLT2i were assessed. Descriptive statistics and logistic regression models were used to examine patient characteristics and treatment patterns.

Results: Among 236 eligible patients, only 16.5%, 10.2%, 31.4%, and 42.8% were receiving target doses of beta-blockers, ACEI/ARB/ARNI, MRA, and SGLT2i, respectively. Quadruple therapy was prescribed in just 21.2% of patients, and fewer than 1% achieved target dosing for all four medication classes. MRA use was strikingly low, with 58.9% of patients not on therapy despite its well-established mortality benefit and relative affordability. Multivariable regression analysis revealed several notable associations. Increasing age was linked to lower odds of ACEI/ARB/ARNI use, while higher systolic blood pressure (SBP) favored treatment. MRA use was more likely among patients with lower SBP, non-ischemic cardiomyopathy, obesity, and absence of atrial fibrillation (AF), with obesity and absence of AF being associated with achieving >50% of the target dose. SGLT2i therapy was more common in younger patients with lower ejection fraction and lower SBP, with type 2 diabetes mellitus being the strongest predictor for both initiation and dose optimization.

Conclusion: These findings highlight a significant gap in GDMT implementation. Despite favorable patient profiles and a high cost-benefit ratio, MRA use remains limited in this cohort. Targeted interventions to support GDMT initiation and optimization in eligible patients may help reduce disparities in heart failure care.

## Introduction

Guideline-directed medical therapy (GDMT) has been the cornerstone for the treatment of patients with heart failure with reduced ejection fraction (HFrEF) for several decades. It includes four core medication classes: evidence-based beta-blockers; angiotensin-converting enzyme inhibitors (ACEI), angiotensin receptor blockers (ARB), or angiotensin receptor-neprilysin inhibitors (ARNI); mineralocorticoid receptor antagonists (MRA); and sodium-glucose co-transporter 2 inhibitors (SGLT2i). When prescribed and titrated to target doses established in landmark clinical trials, these therapies have been shown to significantly reduce mortality and heart failure-related hospitalizations [1,2]. However, despite this robust evidence base, real-world implementation of GDMT remains suboptimal [3,4]. Barriers such as clinical inertia, medication intolerance, socioeconomic constraints, and healthcare disparities, especially in underserved regions, limit consistent utilization of these therapies. To better understand these gaps in care, we conducted an observational study across three outpatient clinics in rural Appalachia, aiming to assess current trends in GDMT adherence and dose optimization among patients with chronic HFrEF.

## Materials and methods

Study design and patient selection

This retrospective observational study included adult patients, 18 years or older, from the East Tennessee State University (ETSU) Health Medical Education Assistance Corporation (MEAC) Altera (previously known as Allscripts) database with a documented diagnosis of HFrEF. Patients were eligible if they had a recorded ejection fraction (EF) ≤ 40% at any time in their medical history and attended at least one follow-up visit between December 1, 2022, and November 30, 2023. Exclusion criteria included those with stage V chronic kidney disease (CKD), those undergoing hemodialysis, baseline serum creatinine >2.5 mg/dL, and serum potassium >5 mmol/L, patients in hospice or comfort care, life expectancy less than one year, heart transplant recipients or those awaiting heart transplant, individuals on left ventricular assist device, or those with contraindications to GDMT components including history of angioedema, recurrent genitourinary infections (two or more in six months or three or more in one year), second- or third-degree atrioventricular block, sick sinus syndrome, severe bradycardia (heart rate <45 beats/minute), significant first-degree heart block with P-R interval ≥0.24 seconds (except in patients with a functioning artificial pacemaker), and unacceptable side effects or hypersensitivity to any of the GDMT.

Demographic and clinical variables collected included age, sex, lowest and most recent EF, systolic and diastolic blood pressure, heart rate, serum potassium, estimated glomerular filtration rate (eGFR), hemoglobin A1c, and baseline serum creatinine. Relevant medical history included etiology of cardiomyopathy, coronary artery disease, peripheral artery disease with prior foot amputation, atrial fibrillation (AF), hypertension, type 2 diabetes mellitus, obstructive sleep apnea, asthma, chronic obstructive pulmonary disease (COPD), active smoking status, use of cardiac devices like implantable cardioverter defibrillator (ICD)/cardiac resynchronization therapy (CRT), and diuretic therapy if any. All data were de-identified. The ETSU Institutional Review Board approved the study. No external funding was received for this study. The design, data collection, analysis, interpretation, and writing were conducted independently by the authors. AI tools were utilised for checking grammatical errors in the final write-up.

Baseline medication data

We assessed baseline use and dosing of the four GDMT medication classes (evidence-based beta-blocker, ACEI/ARB/ARNI, MRA, and SGLT2i). Patients with documented contraindications or intolerances were excluded from the analysis. For each drug class, doses were categorized as none, <50%, ≥50 to <100%, and 100% of the target dose as defined by the 2022 clinical practice guidelines for management of heart failure [1].

Statistical analysis

An initial sample size of 243 patients was reduced to 236 after exclusion of individuals who did not meet the eligibility criteria. Data processing and analysis were conducted in SPSS version 29 (IBM Corp., Armonk, NY, USA), SAS 9.4 (SAS Institute Inc., Cary, NC, USA), and Microsoft Excel 2025 (Redmond, WA, USA). EF values reported as a range were averaged, missing data were replaced with blanks, and medications were re-coded into binary and ordinal categories based on dosing level. A composite GDMT score was created (range: zero to four) based on the number of drug classes prescribed. A score of one indicated monotherapy, and a score of four indicated complete quadruple therapy.

Descriptive statistics included all participant characteristics, each medication class, and their respective target doses, patient demographics, clinical characteristics, and medical history. Continuous patient characteristics were reported as medians with interquartile ranges, while categorical variables were reported as frequencies and percentages. Unadjusted and adjusted logistic regression models were used to explore the predictors of achieving >50% of the target dose for evidence-based beta-blockers and ACEI/ARB/ARNI, as well as to assess predictors of achieving any dose or full dose for MRA and SGLT2i. 

Backward selection criteria with p-value ≤ 0.2 and Wald chi-square test (χ2) were used to determine the independent contribution of each participant’s characteristics to the outcome measure and final model inclusion. Using the likelihood ratio (LR), a stepwise selection procedure, a 95% confidence interval (CI) with alpha set at 0.05, the independent and combined contributions of each participant’s characteristics to the outcome measures were determined. Non-significant characteristics were eliminated using backward selection, starting with the highest p-value. Model fit estimates between the full and reduced models were compared using established criteria (LR test, Hosmer-Lemeshow goodness-of-fit test, AIC, R-squared, and p-value). Lastly, the level of missing data was assessed and accounted for appropriately. Results of logistic regression analyses were presented as odds ratios, 95% CI, and p-value <0.05. Statistical analysis was conducted between October 2024 and February 2025.

## Results

Among the 236 patients included in the study, the median age was 65 years, and 89 (37.7%) were female. The median lowest recorded EF was 27.9%, which improved to a median of 38.4% at the most recent follow-up. Comorbidities were common: hypertension was present in 204 (86.4%), type 2 diabetes mellitus in 89 (37.9%), and coronary artery disease in 160 (67.8%) patients (Table 1). Utilization and optimization of GDMT for HFrEF varied considerably across medication classes. Evidence-based beta-blockers were prescribed in 217 (91.9%) patients, but only 39 (16.5%) of them reached the target dose. Similarly, while 210 (89.0%) patients received an ACEI, ARB, or ARNI, just 24 (10.2%) were on full dosing. MRAs were significantly underutilized, with 139 (58.9%) patients not receiving therapy and only 74 (31.4%) achieving the target dose. SGLT2 inhibitors were prescribed to 105 (44.5%) patients, and 101 (42.8%) reached full target dosing. In terms of overall GDMT intensity, 25 (10.6%) patients were on monotherapy, 79 (33.5%) on dual therapy, 82 (34.8%) on triple therapy, and only 50 (21.2%) received all four recommended drug classes. Strikingly, just two (0.8%) patients were on quadruple therapy at full target doses. These findings highlight substantial gaps in both the initiation and optimization of evidence-based therapies for patients with HFrEF.

**Table 1:** Baseline characteristics of entire population included in the guideline-directed medical therapy study Continuous values are reported as median (quantile 1, quantile 3); Categorical variables are reported as frequencies and (%). Ejection fraction (EF) reported as a percentage average. Lowest EF refers to the EF ≤ 40% recorded anytime during the lifetime of the patient; Latest EF refers to EF recorded during the timeline of conducting the study; Serum potassium is recorded as the average of the last three readings; Active cigarette smoking refers to current cigarette use or smoking cessation within five years. ACEI = angiotensin converting enzyme inhibitor; ARB = angiotensin receptor blocker; ARNI = angiotensin receptor/neprilysin inhibitor; MRA = mineralocorticoid receptor antagonist; SGLT2i = sodium-glucose co-transporter 2 inhibitor; BMI = body mass index. *Hemoglobin A1c data was only available for 147 patients.

Variables	N = 236	%
Age, years	65.4 (13.6)	
Female	89	37.7
Male	147	62.3
Lowest ejection fraction, %	27.9 (7.9)	
Latest ejection fraction, %	38.4 (12.5)	
Vital signs and laboratory findings		
Systolic blood pressure, mmHg	126 (20)	
Diastolic blood pressure, mmHg	75 (12)	
Heart rate, beats/min	77 (14)	
Serum potassium, mmol/L	4.2 (0.4)	
eGFR, ml/min per 1.73m^2^		
15-29	7	3.0
30-59	66	28.1
60-89	100	42.6
≥ 90	62	26.4
Hemoglobin A1c, % *	6.8 (1.7)	
Obesity, BMI ≥30kg/m^2^	106	45.1
Baseline serum creatinine mg/dL	1.1 (0.3)	
Medical history		
Ischemic cardiomyopathy	134	57.5
Nonischemic cardiomyopathy	99	42.5
Coronary artery disease	160	67.8
Foot amputation from peripheral artery disease	92	39.5
Atrial fibrillation	92	39.5
Hypertension	204	86.4
Type 2 diabetes mellitus	89	37.9
Obstructive sleep apnea	68	28.8
Asthma/ chronic obstructive pulmonary disease (COPD)	77	32.6
Active cigarette smoking	111	47.4
Implantable cardioverter defibrillator (ICD) or cardiac resynchronization therapy (CRT)	78	33.1
Categories of furosemide doses, mg		
None	115	48.7
Any dose (≥1)	121	51.3
Target dosing achieved by drug class		
Evidence-based beta-blockers		
None	19	8.1
<50%	130	55.1
≥50% - <100%	48	20.3
100%	39	16.5
ACEI/ARB/ARNI		
None	26	11.0
<50%	138	58.5
≥50% - <100%	48	20.3
100%	24	10.2
MRA		
None	139	58.9
<50%	0	0
≥50% - <100%	23	9.8
100%	74	31.4
SGLT2i		
None	131	55.5
<50%	0	0
≥50% - <100%	4	1.7
100%	101	42.8
Combination therapy		
Monotherapy	25	10.6
Double therapy	79	33.5
Triple therapy	82	34.8
Quadruple therapy	50	21.2

Evidence-based beta-blockers

Baseline clinical characteristics of patients on evidence-based beta-blocker therapy, stratified by dosing, are outlined in Table 2. Evidence-based beta-blockers in this analysis include carvedilol, metoprolol succinate, and bisoprolol. These medications were widely used in the cohort, with only 19 (8.1%) patients not receiving any beta-blocker therapy. Of those treated, 130 (55.1%) patients received less than half of the recommended target dose, 48 (20.3%) received more than half but less than the full target dose, and only 39 (16.5%) achieved the full target dose. This indicates that, while the initiation rates are high, optimization to the target doses remains a significant challenge.

**Table 2:** Baseline patient characteristics by dose of evidence-based beta-blocker therapy Continuous values are reported as median (quantile 1, quantile 3); Categorical variables are reported as frequencies and (%). Ejection fraction (EF) is reported as a percentage average. Lowest EF refers to the EF ≤ 40% recorded any time during the lifetime of the patient; Latest EF refers to EF recorded during the timeline of conducting the study. Serum potassium is recorded as the average of the last three readings; Active cigarette smoking refers to current cigarette use or smoking cessation within five years. PAD = peripheral artery disease; COPD = chronic obstructive pulmonary disease; BMI = body mass index; ICD = implantable cardioverter defibrillator; CRT = cardiac resynchronization therapy; eGFR = estimated glomerular filtration rate. * There was one participant with missing data for eGFR; three participants with missing data for etiology of cardiomyopathy; one participant with missing data for obesity status; three participants with missing data for atrial fibrillation; one participant with missing data for peripheral artery disease; one participant with missing data for type 2 diabetes mellitus; two participants with missing data for active smoking. For hemoglobin A1c, there are 13 participants with missing data for ≥50% target dose, 60 participants with missing data for <50% target dose, and four participants with missing data for none.

Variables	None (n = 19)	<50% target dose (n = 130)	≥50% but <100% target dose (n = 48)	100% target dose (n = 39)
Age, years	64 (60-79)	66 (56-76)	68 (55-75)	66 (49-74)
Sex, n (%)				
Female	3 (15.8)	49 (37.7)	18 (37.5)	19 (48.7)
Male	16 (84.2)	81 (62.3)	30 (62.5)	20 (51.3)
Lowest ejection fraction, %	30 (23-35)	28 (23-35)	28 (21-33)	25 (20-38)
Latest ejection fraction, %	43 (33-53)	38 (28-48)	38 (28-48)	38 (28-48)
Vital signs and laboratory findings				
Systolic blood pressure, mmHg	119 (113-136)	124 (111-137)	126 (117-143)	125 (114-138)
Diastolic blood pressure, mmHg	75 (71-86)	74 (67-82)	76 (67-83)	73 (69-85)
Heart rate, beats/min	79 (70-95)	76 (67-84)	73 (66-86)	80 (70-92)
Serum potassium, mmol/L	4.3 (4.0-4.5)	4.2 (4.0-4.4)	4.2 (3.8-4.4)	4.3 (4.1-4.5)
eGFR, ml/min per 1.73m2*				
15-29	0 (0.0)	5 (3.9)	1 (2.1)	1 (2.6)
30-59	4 (21.1)	38 (29.5)	10 (20.8)	14 (35.9)
60-89	11 (57.9)	49 (38.0)	24 (50.0)	16 (41.0)
≥ 90	4 (21.1)	37 (28.7)	13 (27.1)	8 (20.5)
Hemoglobin A1c, %*	5.7 (5.4-7.1)	6.3 (5.7-7.2)	6.2 (5.7-7.2)	6.5 (5.9-8.0)
Obesity, BMI ≥30kg/m2*	9 (47.4)	49 (38.0)	24 (50.0)	24 (61.5)
Baseline serum creatinine mg/dL	1.02 (0.90-1.20)	1.00 (0.87-1.20)	0.96 (0.80-1.20)	1.09 (0.90-1.30)
Medical history				
Ischemic cardiomyopathy*	9 (47.4)	82 (63.6)	25 (53.2)	18 (47.4)
Non-ischemic cardiomyopathy*	10 (52.6)	47 (36.4)	22 (46.8)	20 (52.6)
Coronary artery disease	11 (57.9)	92 (70.8)	31 (64.6)	26 (66.7)
Foot amputation from PAD*	1 (5.3)	18 (14.0)	6 (12.5)	11 (28.2)
Atrial fibrillation*	13 (68.4)	46 (35.9)	18 (37.5)	15 (39.5)
Hypertension	16 (84.2)	107 (82.3)	45 (93.8)	36 (92.3)
Type 2 diabetes mellitus*	7 (36.8)	43 (33.3)	19 (39.6)	20 (51.3)
Obstructive sleep apnea	9 (47.4)	26 (20.0)	18 (37.5)	15 (38.5)
Asthma/COPD	10 (52.6)	38 (29.2)	13 (27.1)	16 (41.0)
Active cigarette smoking*	5 (27.8)	64 (49.6)	19 (39.6)	23 (59.0)
ICD/CRT	5 (26.3)	42 (32.3)	10 (20.8)	21 (53.9)
Categories of furosemide doses, mg				
None	7 (36.8)	67 (51.5)	28 (58.3)	13 (33.3)
Any dose (≥1)	12 (63.2)	63 (48.5)	20 (41.7)	26 (66.7)

Patients not on beta-blockers were slightly younger and predominantly male compared to those on target dosing. Vital signs, serum potassium, and renal function were similar across groups, suggesting that these factors were not major barriers to use or titration. Obesity was more prevalent in patients on higher doses. Device therapy (ICD/CRT) was most common among those at target doses, possibly reflecting more advanced disease requiring comprehensive management. Additionally, the presence of a device with backup pacing capabilities may have provided clinicians with greater confidence in up-titrating beta-blockers, mitigating concerns over symptomatic bradycardia during dose escalation.

Angiotensin converting enzyme inhibitors, angiotensin receptor blockers, or angiotensin receptor-neprilysin inhibitors 

ACEI, ARB, and ARNI exert their therapeutic benefit in HFrEF by inhibiting various components of the renin-angiotensin-aldosterone system (RAAS). Table 3 summarizes patient characteristics by ACEI/ARB/ARNI dosing. While overall use of this medication class was high, dose optimization was limited as 138 (58.5%) patients received <50% of the target dose, 48 (20.3%) were on ≥50% dose but below target, and only 24 (10.2%) reached full dosing. This indicates that despite strong initiation rates, a substantial gap in dose titration persists, as nearly 70% of patients remain on subtherapeutic doses.

**Table 3:** Baseline patient characteristics by dose of ACEI/ARB/ARNI therapy Continuous values are reported as median (quantile 1, quantile 3); Categorical variables are reported as frequencies and (%). ACEI = angiotensin converting enzyme inhibitor; ARB = angiotensin receptor blocker; ARNI = angiotensin receptor neprilysin inhibitor; PAD = peripheral artery disease; COPD = chronic obstructive pulmonary disease; BMI = body mass index; ICD = implantable cardioverter defibrillator; CRT = cardiac resynchronization therapy; eGFR = estimated glomerular filtration rate. * There was one participant with missing data for eGFR; three participants with missing data for etiology of cardiomyopathy; one participant with missing data for obesity status; three participants with missing data for atrial fibrillation; one participant with missing data for peripheral artery disease; one participant with missing data for type 2 diabetes mellitus; two participants with missing data for active smoking; For Hemoglobin A1c, there are seven participants with missing data for 100% target dose, 22 participants with missing data for ≥50% target dose, 49 participants with missing data for <50% target dose, and 11 participants with missing data for none.

Variables	None (n = 26)	<50% target dose (n = 138)	≥50% but <100% target dose (n = 48)	100% target dose (n = 24)
Age, years	73 (64-86)	68 (57-77)	63 (53-70)	61 (50-73)
Female	11 (42.3)	48 (34.8)	20 (41.7)	10 (41.7)
Male	15 (57.7)	90 (65.2)	28 (58.3)	14 (58.3)
Lowest ejection fraction, %	28 (20-35)	28 (23-34)	30 (23-38)	28 (21-36)
Latest ejection fraction, %	34 (25-49)	38 (28-48)	41 (30-50)	41 (28-48)
Vital signs and laboratory findings				
Systolic blood pressure, mmHg	115 (110-136)	122 (111-135)	135 (116-150)	130 (121-138)
Diastolic blood pressure, mmHg	72 (68-81)	74 (67-82)	78 (73-84)	74 (67-86)
Heart rate, beats/min	80 (65-88)	77 (68-87)	73 (66-83)	77 (70-89)
Serum potassium, mmol/L	4.2 (3.9-4.3)	4.2 (4.0-4.5)	4.3 (3.8-4.4)	4.4 (4.1-4.6)
eGFR, ml/min per 1.73m^2^*				
15-29	0 (0.0)	5 (3.6)	2 (4.3)	0 (0.0)
30-59	10 (38.5)	34 (24.6)	13 (27.7)	9 (37.5)
60-89	10 (38.5)	62 (44.9)	22 (46.8)	6 (25.0)
≥ 90	6 (23.1)	37 (26.8)	10 (21.3)	9 (37.5)
Hemoglobin A1c, %*	5.9 (5.5-7.4)	6.2 (5.7-7.1)	6.5 (6.0-8.9)	6.5 (5.7-7.1)
Obesity, BMI ≥30kg/m^2^*	7 (26.9)	62 (45.3)	23 (47.9)	14 (58.3)
Baseline serum creatinine, mg/dL	1.00 (0.8-1.3)	1.00 (0.88-1.20)	1.00 (0.88-1.30)	1.10 (0.90-1.25)
Medical history				
Ischemic cardiomyopathy*	17 (65.4)	80 (58.8)	25 (52.1)	12 (52.2)
Non-ischemic cardiomyopathy*	9 (34.6)	56 (41.2)	23 (47.9)	11 (47.8)
Coronary artery disease	17 (65.4)	98 (71.0)	29 (60.4)	16 (66.7)
Foot amputation from PAD*	4 (15.4)	18 (13.1)	10 (20.8)	4 (16.7)
Atrial fibrillation*	15 (57.7)	57 (41.6)	13 (28.3)	7 (29.2)
Hypertension	22 (84.6)	117 (84.8)	42 (87.5)	23 (95.8)
Type 2 diabetes mellitus*	9 (34.6)	49 (35.5)	20 (42.6)	11 (45.8)
Obstructive sleep apnea	3 (11.5)	40 (29.0)	15 (31.3)	10 (41.7)
Asthma/ COPD	8 (30.8)	46 (33.3)	14 (29.2)	9 (37.5)
Active cigarette smoking*	10 (38.5)	63 (45.7)	26 (56.5)	12 (50.0)
ICD/CRT	10 (38.5)	51 (37.0)	8 (16.7)	9 (37.5)
Categories of furosemide doses, mg				
None	12 (46.2)	60 (43.5)	29 (60.4)	14 (58.3)
Any dose (≥1)	14 (53.9)	78 (56.5)	19 (39.6)	10 (41.7)

Patients not on therapy were older (median age 73 vs. 61 years at target dose). Systolic blood pressure increased with dose, potentially reflecting clinician caution in those with borderline pressures. Lowest EFs were similar across groups (median 28-30%), but the most recent EF improved with increasing dose, peaking at 41% in patients on ≥50% and 100% target doses, possibly reflecting therapeutic benefits of more aggressive clinical management. Serum potassium and renal function remained stable across groups, suggesting these factors were not major barriers to up-titration. AF was notably more prevalent in untreated patients and decreased with increasing dose. Loop diuretic use was highest among untreated patients and less common in those receiving higher doses, possibly reflecting better volume control with optimized RAAS blockade.

Mineralocorticoid receptor antagonists

Table 4 highlights significant underutilization of MRA in this HFrEF cohort, as 139 (58.9%) patients were not on therapy, 23 (9.7%) were on ≥50% but sub-target dosing, and only 74 (31.4%) reached the target dose. Patients not on MRAs were older (median age 69 vs. 62 years at target dose). Notably, those not receiving MRAs had a higher median lowest EF (33%) compared to those on full or partial dosing (23%). Blood pressure, potassium, and renal function were generally comparable across groups. Notably, most untreated patients had preserved kidney function, indicating missed opportunities for initiation. Obesity, diabetes, and device therapy were more common in patients at target doses, reflecting more advanced disease. 

**Table 4:** Baseline patient characteristics by dose of MRA therapy No participants are taking <50% of the recommended target dose of MRA, hence these categories were excluded. Continuous values are reported as median (quantile 1, quantile 3); Categorical variables are reported as frequencies and (%). MRA = mineralocorticoid receptor antagonist; eGFR = estimated glomerular filtration rate; PAD = peripheral artery disease; COPD = chronic obstructive pulmonary disease; BMI = body mass index; ICD = implantable cardioverter defibrillator; CRT = cardiac resynchronization therapy. * There was one participant with missing data for eGFR; three participants with missing data for etiology of cardiomyopathy; one participant with missing data for obesity status; three participants with missing data for atrial fibrillation; one participant with missing data for peripheral artery disease; one participant with missing data for type 2 diabetes mellitus; two participants with missing data for active smoking; For Hemoglobin A1c, there are 23 participants with missing data for 100% target dose, 11 participants with missing data for ≥50% target dose, and 55 participants with missing data for none.

Variables	None (n = 139)	≥50% but <100% target dose (n = 23)	100% target dose (n = 74)
Age, years	69 (59-77)	65 (54-77)	62 (52-73)
Female	51 (36.7)	10 (43.5)	28 (37.8)
Male	88 (63.3)	13 (56.5)	46 (62.2)
Lowest ejection fraction, %	33 (23-35)	23 (20-35)	23 (18-33)
Latest ejection fraction, %	40 (33-49)	38 (23-45)	36 (25-45)
Vital signs and laboratory findings			
Systolic blood pressure, mmHg	127 (113-142)	120 (109-127)	123 (111-136)
Diastolic blood pressure, mmHg	74 (68-83)	72 (65-80)	76 (67-84)
Heart rate, beats/min	76 (67-86)	82 (63-88)	76 (69-86)
Serum potassium, mmol/L	4.2 (3.9-4.4)	4.3 (4.0-4.5)	4.3 (4.0-4.5)
eGFR, ml/min per 1.73m^2^*			
15-29	7 (5.1)	0 (0.0)	0 (0.0)
30-59	38 (27.5)	7 (30.4)	21 (28.4)
60-89	58 (42.0)	12 (52.2)	30 (40.5)
≥ 90	35 (25.4)	4 (17.4)	23 (31.1)
Hemoglobin A1c, %*	6.2 (5.7-7.3)	6.3 (5.4-8.7)	6.3 (5.7-7.2)
Obesity, BMI ≥30kg/m^2^*	53 (38.4)	12 (52.2)	41 (55.4)
Baseline serum creatinine, mg/dL	1.00 (0.86-1.30)	1.00 (0.90-1.44)	1.00 (0.89-1.20)
Medical history			
Ischemic cardiomyopathy*	85 (62.5)	8 (34.8)	41 (55.4)
Non-ischemic cardiomyopathy*	51 (37.5)	15 (65.2)	33 (44.6)
Coronary artery disease	95 (68.4)	13 (56.5)	52 (70.3)
Foot amputation from PAD*	17 (12.3)	1 (4.4)	18 (24.3)
Atrial fibrillation*	64 (46.7)	9 (39.1)	19 (26.0)
Hypertension	120 (86.3)	17 (73.9)	67 (90.5)
Type 2 diabetes mellitus*	52 (37.7)	5 (21.7)	32 (43.2)
Obstructive sleep apnea	39 (28.1)	7 (30.4)	22 (29.7)
Asthma/COPD	47 (33.8)	6 (26.1)	24 (32.4)
Active cigarette smoking*	65 (47.1)	8 (34.8)	38 (52.1)
ICD/CRT	38 (27.3)	9 (39.1)	31 (41.9)
Categories of furosemide doses, mg			
None	76 (54.7)	10 (43.5)	29 (39.2)
Any dose (≥1)	63 (45.3)	13 (56.5)	45 (60.8)

Sodium-glucose co-transporter 2 inhibitors

Table 5 outlines baseline characteristics by SGLT2i dosing. Of the 236 patients, 101 (42.8%) were on target doses, four (1.7%) on ≥50% but sub-target doses, and 131 (55.5%) were not receiving therapy. Patients not on SGLT2i were older (median 69 years vs 64 years at target dose). Sex distribution was similar across groups. Compared to non-users, patients on target doses of SGLT2i had lower median lowest EF (23% vs. 30%). Metabolic comorbidities were notably more prevalent among the treated patient group, with 55 (51%) individuals presenting with obesity and 58 (54.5%) diagnosed with type 2 diabetes mellitus. Despite evidence of benefit regardless of glycemic status, the relatively low uptake in non-diabetics underscores a lingering gap in the broader application of SGLT2i therapy. Treated patients also had higher rates of ischemic cardiomyopathy, coronary disease, and device therapy. Loop diuretic use was more common in non-users, while 65 (61.9%) patients on SGLT2i were not on diuretics, suggesting SGLT2i may be viewed as an alternative, and not just an adjunct in volume management.

**Table 5:** Baseline patient characteristics by dose of SGLT2i therapy No participants are taking <50% of the recommended target dose of SGLT2i, hence these categories were excluded. Continuous values are reported as median (quartile 1, quartile 3); Categorical variables are reported as frequencies and (%). SGLT2i = Sodium glucose co-transporter 2 inhibitor; PAD = peripheral artery disease; COPD = chronic obstructive pulmonary disease; BMI = body mass index; ICD = implantable cardioverter defibrillator; CRT = cardiac resynchronization therapy; eGFR = estimated glomerular filtration rate. * There was one participant with missing data for eGFR; three participants with missing data for etiology of cardiomyopathy; one participant with missing data for obesity status; three participants with missing data for atrial fibrillation; one participant with missing data for peripheral artery disease; one participant with missing data for type 2 diabetes mellitus; two participants with missing data for active smoking; For hemoglobin A1c, there are 24 participants with missing data for 100% target dose, one participant with missing data for ≥50% target dose, and 64 participants with missing data for none.

Variables	None (n = 131)	≥50% but <100% target dose (n = 4)	100% target dose (n = 101)
Age, years	69 (57-78)	80 (72-84)	64 (52-72)
Female	49 (37.4)	1 (25.0)	39 (38.6)
Male	82 (62.6)	3 (75.0)	62 (61.4)
Lowest ejection fraction, %	30 (23-38)	35 (29-38)	23 (20-33)
Latest ejection fraction, %	40 (33-48)	44 (33-50)	38 (28-48)
Vital signs and laboratory findings			
Systolic blood pressure, mmHg	129 (114-142)	129 (112-145)	121 (110-134)
Diastolic blood pressure, mmHg	74 (68-82)	74 (67-97)	75 (67-84)
Heart rate, beats/min	75 (67-85)	71 (64-74)	79 (69-89)
Serum potassium, mmol/L	4.2 (3.9-4.4)	4.0 (3.7-4.6)	4.3 (4.0-4.5)
eGFR, ml/min per 1.73m^2^*			
15-29	3 (2.3)	1 (25.0)	3 (3.0)
30-59	33 (25.2)	2 (50.0)	31 (31.0)
60-89	60 (45.8)	0 (0.0)	40 (40.0)
≥ 90	35 (26.7)	1 (25.0)	26 (26.0)
Hemoglobin A1c, %*	6.0 (5.4-6.3)	6.0 (5.5-7.6)	6.7 (5.9-8.0)
Obesity, BMI ≥30kg/m^2^*	54 (41.2)	1 (25.0)	51 (51.0)
Baseline serum creatinine, mg/dL	1.00 (0.86-1.20)	1.15 (0.91-1.80)	1.00 (0.90-1.20)
Medical history			
Ischemic cardiomyopathy*	70 (53.9)	3 (100.0)	61 (61.0)
Non-ischemic cardiomyopathy*	60 (46.2)	0 (0.0)	39 (39.0)
Coronary artery disease	84 (64.1)	4 (100.0)	72 (71.3)
Foot amputation from PAD*	18 (13.9)	1 (25.0)	17 (16.8)
Atrial fibrillation*	57 (43.9)	2 (50.0)	33 (33.3)
Hypertension	114 (87.0)	3 (75.0)	87 (86.1)
Type 2 diabetes mellitus*	31 (23.9)	3 (75.0)	55 (54.5)
Obstructive sleep apnea	35 (26.7)	2 (50.0)	31 (30.7)
Asthma/COPD	43 (32.8)	2 (50.0)	32 (31.7)
Active cigarette smoking*	61 (47.3)	0 (0.0)	50 (49.5)
ICD/CRT	40 (30.5)	0 (0.0)	38 (37.6)
Categories of furosemide doses, mg			
None	56 (42.8)	3 (75.0)	62 (61.4)
Any dose (≥1)	75 (57.3)	1 (25.0)	39 (38.6)

Multivariable regression analyses

Multivariable regression analysis (Table 6) revealed several clinical factors that were associated with the likelihood of treatment and dose optimization of GDMT. For ACEI/ARB/ARNI, increasing age was linked to significantly lower odds of receiving >50% dose (p = 0.015), whereas higher systolic blood pressure favored treatment with higher doses (p = 0.011), indicating that hypotension may be a limiting factor, particularly in older adults. In contrast, MRA use was more likely in patients with lower systolic blood pressure (p = 0.02), non-ischemic cardiomyopathy (p < 0.05), obesity (p = 0.03), and absence of AF (p = 0.012). Notably, both obesity (p < 0.05) and absence of AF (p = 0.009) were also predictors of achieving 100% of the target dose for MRA. SGLT2i therapy was more commonly initiated in younger patients (p = 0.04), those with lower ejection fraction (p < 0.05), and lower systolic blood pressure (p = 0.018), with type 2 diabetes mellitus emerging as the strongest predictor for both treatment initiation and optimization to target dose (both p < 0.001). 

**Table 6:** Multivariable logistic regression of guideline-directed medical therapy drug doses and participant characteristics Ejection fraction (EF), originally collected as a percentage range, is now reported as a percentage average. The latest EF refers to EF recorded during the study's timeline. COPD = chronic obstructive pulmonary disease; BMI = body mass index; ACEI = angiotensin converting enzyme inhibitor; ARB = angiotensin receptor blocker; ARNI = angiotensin receptor/neprilysin inhibitor; MRA = mineralocorticoid receptor antagonist; SGLT2i = sodium-glucose co-transporter 2 inhibitor; Continuous variables are presented as a 10-fold increase. Statistical significance with  P<0.05.

Participant characteristics	Treated (any dose) vs. not treated (none)	>50% target Dose vs. <50% target Dose	100% target dose vs. <100% target dose
Evidence-based beta-blockers			
Age, per 10-year increase	-	0.86 (0.70, 1.06), p=0.167	-
Systolic blood pressure, per 10-mmHg increase	-	1.11 (0.97, 1.28), p=0.133	-
Sex: female	-	1.35 (0.76, 2.37), p=0.30	-
Cardiomyopathy: ischemic	-	0.60 (0.34, 1.07), p=0.083	-
BMI status: obesity	-	1.54 (0.87, 2.73), p=0.138	-
Type 2 diabetes mellitus	-	1.70 (0.95, 3.06), p=0.076	-
ACEI/ARB/ARNI			
Age, per 10-year increase	-	0.71 (0.54, 0.94), p=0.015	-
Systolic blood pressure, per 10-mmHg increase	-	1.23 (1.05, 1.43), p=0.011	-
Sex: female	-	1.30 (0.70, 2.44), p=0.40	-
BMI status: obesity	-	1.31 (0.70, 2.47), p=0.40	-
Coronary artery disease	-	0.94 (0.48, 1.84), p=0.86	-
Atrial fibrillation	-	0.72 (0.36, 1.44), p=0.35	-
Active cigarette smoking	-	1.09 (0.56, 2.11), p=0.80	-
Categories of furosemide doses, mg any dose (≥1)	-	0.56 (0.30, 1.04), p=0.066	-
MRA			
Age, per 10-year increase	0.88 (0.69, 1.11), p=0.27	-	0.91 (0.71, 1.16), p=0.43
Latest ejection fraction, per absolute 10% increase	0.76 (0.60, 0.98), p=0.030	-	0.81 (0.63, 1.05), p=0.107
Systolic blood pressure, per 10-mmHg increase	0.83 (0.71, 0.97), p=0.021	-	0.92 (0.79, 1.08), p=0.32
Sex: female	1.06 (0.57, 1.96), p=0.85	-	0.87 (0.46, 1.67), p=0.68
Cardiomyopathy: ischemic	0.55 (0.30, 0.99), p=0.047	-	
BMI status: obesity	1.95 (1.07, 3.53), p=0.028	-	1.86 (1.00, 3.43), p=0.048
Atrial fibrillation	0.42 (0.22, 0.82), p=0.012	-	0.39 (0.20, 0.79), p=0.009
Categories of furosemide doses, mg any dose (≥1)	1.61 (0.87, 2.97), p=0.127	-	1.86 (0.98, 3.54), p=0.058
SGLT2i			
Age, per 10-year increase	0.77 (0.60, 0.99), p=0.044	-	0.74 (0.57, 0.95), p=0.019
Latest ejection fraction, per absolute 10% increase	0.77 (0.60, 0.99), p=0.045	-	0.76 (0.59, 0.98), p=0.035
Systolic blood pressure, per 10-mmHg increase	0.83 (0.71, 0.97), p=0.018	-	0.82 (0.70, 0.96), p=0.011
Heart rate, per 10-beats/min increase	0.93 (0.74, 1.17), p=0.52	-	0.97 (0.77, 1.21), p=0.76
Sex: female	1.28 (0.68, 2.41), p=0.44	-	1.31 (0.70, 2.46), p=0.40
Cardiomyopathy: ischemic	1.22 (0.52, 2.87), p=0.65	-	
BMI status: obesity	1.06 (0.57, 1.97), p=0.87	-	1.20 (0.65, 2.22), p=0.57
Coronary artery disease	0.90 (0.36, 2.26), p=0.82	-	
Atrial fibrillation	0.63 (0.31, 1.26), p=0.192	-	0.68 (0.34, 1.33), p=0.26
Type 2 diabetes mellitus	5.29 (2.73, 10.25), p=<0.001	-	4.72 (2.50, 8.90), p=<0.001
Categories of furosemide doses, mg any dose (≥1)	2.25 (1.17, 4.33), p=0.015	-	2.01 (1.06, 3.83), p=0.034

Combination therapy

Table 7 reveals the hierarchy of initiation of GDMT across different stages of heart failure treatment, as reflected by patients on monotherapy, double, triple, and quadruple therapy. Among patients on monotherapy, beta-blockers were most commonly initiated, highlighting their role as the primary starting point in treatment. As patients progressed to double and triple therapy, the addition of ACEI/ARB/ARNI became more frequent, demonstrating their position as the next step in therapy escalation. MRA and SGLT2i were less commonly used in monotherapy and double therapy groups but became increasingly incorporated in triple and quadruple therapy regimens. This distribution indicates a clear stepwise approach to initiating heart failure treatments, starting predominantly with beta-blockers, followed by ACEI/ARB/ARNI, and then adding MRA and SGLT2i as therapy intensifies.

**Table 7:** Distribution of participants by guideline-directed medical therapy combinations and achieved dosing ACEI = angiotensin converting enzyme inhibitor; ARB = angiotensin receptor blocker; ARNI = angiotensin receptor-neprilysin inhibitor; MRA = mineralocorticoid receptor antagonist; SGLT2i = sodium glucose co-transporter 2 inhibitor. No participants are taking <50% of the recommended target dose of MRA and SGLT2i, hence these categories were excluded.

Drug category by class and dose (N = 236)	Monotherapy (N=25)	Double therapy (N=79)	Triple therapy (N=82)	Quadruple therapy (N=50)
Beta-blocker	n (%)	n (%)	n (%)	n (%)
None (n=19)	11 (44.0)	7 (8.9)	1 (1.2)	0 (0.0)
<50% (n=130)	10 (40.0)	48 (60.8)	46 (56.1)	26 (52.0)
≥50% - <100% (n=48)	2 (8.0)	19 (24.1)	16 (19.5)	11 (22.0)
100% (n=39)	2 (8.0)	5 (6.3).	19 (23.2)	13 (26.0)
ACEI/ARB/ARNI				
None (n=26)	15 (60.0)	10 (12.7)	1 (1.2)	0 (0.0)
<50% (n=138)	8 (32.0)	41 (51.9)	58 (70.7)	31 (62.0)
≥50% - <100% (n=48)	1 (4.0)	20 (25.3)	16 (19.5)	11 (22.0)
100% (n=24)	1 (4.0)	8 (10.1)	7 (8.5)	8 (16.0)
MRA				
None (n=139)	25 (100.0)	72 (91.1)	42 (51.2)	0 (0.0)
≥50% - <100% (n=23)	0 (0.0)	3 (3.8)	7 (8.5)	13 (26.0)
100% (n=74)	0 (0.0)	4 (5.1)	33 (40.2)	37 (74.0)
SGLT2 inhibitor				
None (n=131)	24 (96.0)	69 (87.3)	38 (46.3)	0 (0.0)
≥50% - <100% (n=4)	0 (0.0)	1 (1.3)	2 (2.4)	1 (2.0)
100% (n=101)	1 (4.0)	9 (11.4)	42 (51.2)	49 (98.0)

## Discussion

To contextualize our findings, it is important to review the foundational clinical trials that have shaped GDMT in HFrEF. ACEI were the first RAAS inhibitors shown to reduce mortality in HFrEF, as demonstrated in the CONSENSUS trial published in 1987 [5]. The ELITE II study showed that losartan was non-inferior to captopril in reducing cardiovascular morbidity and mortality, while the CHARM-Alternate trial demonstrated that candesartan was effective in achieving the same end points in those intolerant to ACEI compared to placebo [6,7]. More recently, the PARADIGM-HF trial established the superiority of ARNI, specifically sacubitril-valsartan, over enalapril in reducing cardiovascular mortality and rehospitalizations [8]. Although generally well tolerated, RAAS inhibitors carry risks of acute kidney injury, hyperkalemia, and hypotension. ACEI and ARNI can also cause cough and, rarely, angioedema [9-11]. Beta-blockers remain integral to the management of HFrEF. However, only metoprolol succinate, carvedilol, and bisoprolol have proven mortality benefits. The MERIT-HF and COMET trials support the efficacy of metoprolol succinate and carvedilol, with carvedilol showing superiority over metoprolol, likely due to its additional alpha-blocking effects. Common side effects include hypotension and bradycardia, and use is often limited in patients with pulmonary comorbidities due to concern for bronchospasm [1,12-14]. MRAs like spironolactone and eplerenone have demonstrated additional mortality benefits in patients with HFrEF already stabilized on standard therapy of ACEI, beta-blockers, and diuretics. Their use is supported by trials such as RALES and EMPHASIS-HF [15,16]. Side effects include acute kidney injury, hyperkalemia, and, with spironolactone, gynecomastia - less common with eplerenone due to its selectivity to aldosterone receptors [1,15]. Lastly, the use of SGLT2i represents a major advancement in the treatment of HFrEF. Landmark trials such as DAPA-HF and EMPEROR-Reduced demonstrated that dapagliflozin and empagliflozin, respectively, significantly reduce the risk of cardiovascular death and heart failure hospitalization when added to standard therapy, regardless of the patient’s diabetic status [17,18]. SGLT2i should be used with caution in patients with recurrent genitourinary infections [1,17,18].

Despite the well-researched benefits of GDMT for HFrEF, its implementation in real-world clinical practice remains suboptimal [3,4]. In our study, we assessed the medication regimens of stable HFrEF patients receiving care in the outpatient setting in rural Appalachia, a region often characterized by limited healthcare access and socioeconomic challenges. After carefully excluding patients with documented contraindications to components of GDMT, we found that only 50 (21.2%) of the study population were prescribed all four recommended classes of GDMT, with less than 1% of the entire cohort receiving target doses of quadruple therapy. We found the most common regimen was triple therapy, followed by double therapy. In patients receiving triple therapy, the most frequent combination included an ACE/ARB/ARNI, beta-blocker, and SGLT2i. 

In our study, we observed that 139 (58.9%) patients were not prescribed an MRA, despite meeting clinical criteria for its use and excluding all the contraindications. This underutilization is concerning, given the well-established benefits of MRAs in patients with HFrEF, as well as their relative affordability and accessibility in most clinical settings. Spironolactone has been shown to slow heart failure progression by mitigating sodium retention, reducing myocardial and vascular fibrosis, and ultimately lowering the risk of sudden cardiac death [15]. Previously recommended as an adjunct to ACEI/ARB and beta-blockers in patients with HFrEF and EF <35%, MRAs have since been elevated to a Class I recommendation for all patients with EF <40% and New York Heart Association (NYHA) class II to IV symptoms, according to the 2022 AHA/ACC/HFSA guidelines [1,19]. Notably, most patients in this cohort who were not on MRA had preserved renal function (eGFR >60 mL/min/1.73m²), normal potassium levels (~4.2 mmol/L), and adequate blood pressure (median SBP 127 mmHg), suggesting a missed opportunity to improve outcomes. Contributing factors may include clinical inertia, unfamiliarity with evolving guidelines, concerns about adverse effects, or the need for close laboratory monitoring. 

Larger nationwide studies like the CHAnge the Management of Patients with Heart Failure (CHAMP-HF) registry also reported suboptimal use and dosing of GDMT, with only 1% of patients being on target doses of triple therapy with ACEI/ARB/ARNI, beta-blockers, and MRAs. They found a significant underuse of ARNI and MRAs in eligible patients, and reported higher hospitalization rates and worse NYHA functional class in patients receiving the lowest doses of GDMT [3,4]. The Improve the Use of Evidence-Based Heart Failure Therapies in the Outpatient Setting (IMPROVE-HF) study, a large-scale quality improvement initiative published in 2010, demonstrated significant improvement in GDMT utilization with targeted interventions such as clinical decision-making tools, structured improvement strategies, and audit-feedback loops [20,21]. The Acute Decompensated Heart Failure National Registry (ADHERE), Organized Program to Initiate Life-Saving Treatment in Hospitalized Patients With Heart Failure (OPTIMIZE-HF), and Get With The Guidelines-Heart Failure (GWTG-HF) program are US-hospital-based studies aimed at improving the care of patients hospitalized with HF by encouraging the use of process improvement systems and adhering to the latest practice guidelines [22-24].

Limitations

This study has several important limitations. As a retrospective observational analysis, it is inherently subject to selection bias, confounding, and limitations in establishing causality. Medication use and dosing were determined based on documentation in the electronic health record (EHR), which may not fully reflect actual patient adherence or medication changes made outside the health system. Additionally, although patients with documented contraindications to GDMT components were excluded, undocumented clinical considerations such as frailty, hypotension, or patient preference could have influenced prescribing patterns and are not fully accounted for in this analysis. Laboratory and clinical data were limited to values available in the EHR at the time of review. Most clinical and demographic variables in our dataset had very little missing data, typically around one percent. We were unable to account for the heterogeneity of diuretic regimens, including “as-needed” dosing, which may have influenced the interpretation of volume status and medication titration. Lastly, the study sample was drawn from three MEAC-affiliated outpatient Internal Medicine and Cardiology clinics in rural Tennessee, potentially limiting generalizability to other populations. While multivariable modeling was used to adjust for confounding, residual confounding due to unmeasured variables such as socioeconomic status, healthcare access, or clinician-level variation remains possible.

Despite these limitations, to our knowledge, this is the first study to specifically evaluate GDMT prescribing patterns in a rural Appalachian outpatient population. Our findings demonstrate significant underutilization and suboptimal dosing of these evidence-based medications, even among patients who are clinically stable and eligible for therapy.

## Conclusions

This study highlights significant deficiencies in the real-world implementation of GDMT for management of HFrEF in rural Appalachian outpatient settings. Despite eligibility and clinical stability, the majority of patients were not prescribed all four recommended drug classes, and fewer of them achieved target dosing. Underuse of MRAs and suboptimal dosing of beta-blockers and ACEI/ARB/ARNI were particularly evident, even among patients with favorable clinical profiles. These findings underscore a pressing need for targeted quality improvement efforts, clinician education, and system-level interventions to close the gap between evidence and practice. Addressing therapeutic inertia, enhancing clinician familiarity with updated guidelines, and improving access to routine monitoring in rural areas may be helpful in improving outcomes and reducing disparities in heart failure care.
